# A Promising Recombinant Herpesvirus of Turkeys Vaccine Expressing PmpD-N of *Chlamydia psittaci* Based on Elongation Factor-1 Alpha Promoter

**DOI:** 10.3389/fvets.2017.00221

**Published:** 2017-12-14

**Authors:** Shanshan Liu, Wei Sun, Xuefei Huang, Wen Zhang, Changqing Jia, Jie Luo, Yihua Shen, Saeed El-Ashram, Cheng He

**Affiliations:** ^1^Tongren Polytechnic College, Tongren, China; ^2^National and Local Engineering Research Centre for Separation and Purification Ethnic Chinese Veterinary Herbs, Tongren, China; ^3^Key Lab of Animal Epidemiology and Zoonosis of Ministry of Agriculture, College of Veterinary Medicine, China Agricultural University, Beijing, China; ^4^Key Laboratory of Animal Disease and Human Health of Sichuan Province, College of Veterinary Medicine, Sichuan Agricultural University, Wenjiang, China; ^5^School of Life Science and Engineering, Foshan University, Guangdong, China

**Keywords:** *Chlamydia psittaci*, Marek’s disease, herpesvirus of turkeys, PmpD-N, elongation factor-1 alpha promoter

## Abstract

The obligate intracellular Gram-negative bacterium *Chlamydia psittaci* often causes avian chlamydiosis and influenza-like symptoms in humans. However, the commercial subunit *C. psittaci* vaccine could only provide a partial protection against avian chlamydiosis due to poor cellular immune response. In our previous study, a recombinant herpesvirus of turkeys (HVT)-delivered vaccine against *C. psittaci* and Marek’s disease based on human cytomegalovirus (CMV) promoter (rHVT-CMV-*pmpD*) was developed and provided an effective protection against *C. psittaci* disease with less lesions and reduced chlamydial loads. In this study, we developed another recombinant HVT vaccine expressing the N-terminal fragment of PmpD (PmpD-N) based on human elongation factor-1 alpha (EF-1α) promoter (rHVT-EF-*pmpD*) by modifying the HVT genome within a bacterial artificial chromosome. The related characterization of rHVT-EF-*pmpD* was evaluated *in vitro* in comparison with that of rHVT-CMV-*pmpD*. The expression of PmpD-N was determined by western blot. Under immunofluorescence microscopy, PmpD-N protein of both two recombinant viruses was located in the cytoplasm and on the cell surface. Growth kinetics of rHVT-EF-*pmpD* was comparable to that of rHVT-CMV-*pmpD*, and the growth rate of rHVT-EF-*pmpD* was apparently higher than that of rHVT-CMV-*pmpD* on 48, 72, and 120 h postinfection. Macrophages activated by rHVT-EF-*pmpD* could produce more nitric oxide and IL-6 than that activated by rHVT-CMV-*pmpD*. In this study, a recombinant HVT vaccine expressing PmpD-N based on EF-1α promoter was constructed successfully, and a further research *in vivo* was needed to analyze the vaccine efficacy.

## Introduction

*Chlamydia psittaci* is an emerging zoonotic pathogen of global significance, which can cause avian chlamydiosis (1), and infection in human with pneumonia, encephalitis, endocarditis, and even death (2). Study at home, abroad and in our laboratory had indicated that the seropositivity rate of *C. psittaci* was extremely high in poultry flocks (3–6). Currently, no efficacious commercial *C. psittaci* vaccine is available because the cellular and humoral immunity are both necessary to protect animals from this obligate intracellular bacterial infection (7).

The major outer membrane protein (MOMP) and the autotransported polymorphic membrane protein D (PmpD) of *C. psittaci* are proved to be good vaccine candidates (8–10). PmpD has more merit than MOMP because PmpD is conserved and can elicit early immune-mediated neutralization of an ongoing chlamydial infection (11, 12). The specific neutralizing antibody triggered by N-terminal fragment of PmpD (PmpD-N) may provide humoral immune protection against early infection (12).

A herpesvirus of turkeys (HVT) vector-based vaccine can deliver antigens to the surface of cells, and further effectively stimulate cellular immunity and humoral immunity (13). This characteristic is very useful for the development of an effective vaccine for the prevention and control of *Chlamydia* infection (14). Furthermore, a great many of HVT-based recombinant vaccines expressing protective antigens of avian pathogens, such as Newcastle disease virus, infectious bursal disease virus, and highly pathogenic avian influenza, were constructed, and excellent and long-term protective effect in chickens against both pathogens were proved (15–18).

In our previous study, a recombinant HVT expressing PmpD-N based on cytomegalovirus (CMV) promoter (rHVT-CMV-*pmpD*) was constructed to successfully elicit an exceptional cellular and humoral immunity, and finally proved to confer a partial protection in chickens against *C. psittaci* challenge infection (10). In this study, we developed another recombinant HVT vaccine expressing PmpD-N based on elongation factor-1 alpha (EF-1α) promoter (rHVT-EF-*pmpD*), and its morphological and immunological characterization was analyzed in comparison with rHVT-CMV-*pmpD in vitro*.

## Materials and Methods

### Chicken Cells and *C. psittaci* Strain

Primary chicken embryo fibroblast (CEF) cells were prepared from 10-day-old specific-pathogen-free embryos (Vital Merial Experimental Animal Co., Ltd., Beijing, China) (15). The HD11 chicken macrophage cell line was maintained in RPMI-1640 medium with 10% FBS (v/v). The DNA of *C. psittaci* strain CB7 was extracted and stored in our lad as reported previously (10).

### Generation of Recombinant HVT Expressing *pmpD*-N Gene Based on EF-1α Promoter

The recombinant HVT was generated as described previously (10). The recombinant HVT bacterial artificial chromosome (BAC) was based on EF-1α promoter, which was from the vector of pEF6/V5-His (Invitrogen, Carlsbad, CA, USA). A pair of primers used to amplify the expression cassette with the EF-1α promoter at the 5′ end and the BGH polyadenylation (poly A) site at the 3′ end was designed using Oligo 7 (Molecular Biology Insights, USA). The forward primer was 5′-GGTTAATTAACCTTCTAGGTCTTGAAAGGAGTGGGA-3′, and the reverse primer was 5′-GGTTAATTAAGCCTCAGAAGCCATAGAGCCCACC-3′. Both primers contained a *Pac*I restriction site at their 5′ termini. The recombinant virus was designated as rHVT-EF-*pmpD*.

### Plaque Assays and One-Step Growth Kinetics

The plaque size, morphology, and plaque-forming unit (PFU) of rHVT-EF-*pmpD* were compared with those of the same passage of rHVT-CMV-*pmpD* by the immunohistochemical assay as recorded previously (15). Briefly, recombinant viruses were 10-fold diluted and inoculated into the CEF cells, which were seeded in six-well plates. Four days later, ice-clod acetone was added into each well to fix the cells. Cells were washed by phosphate-buffered saline (PBS) and then blocked by blocking buffer (PBS containing 0.1% BSA). Cells were incubated with the wild type HVT-specific polyclonal antibodies raised in chickens (diluted 1:100), and subsequently reacted with horseradish peroxidase-labeled goat-anti-chicken IgG (1:4,000) (Sigma-Aldrich, Shanghai, China). Cells containing the conjugated antibody were identified by incubation at 37°C for 1 h in developing solution containing 1% (w/v) 3-amino-9-ethylcarbazole (Sigma) and 0.02% (v/v) H_2_O_2_ in 0.1 M sodium acetate (pH 4.8). Plaques were counted using an inverted microscope. Moreover, the growth rates of the recombinant viruses were studied on CEF cells by calculating the virus plaques at 12, 24, 48, 72, 96, and 120 h postinfection (16).

### Identification of the Recombinant Viruses Using Immunoblot and Immunofluorescence

Immunoblot analysis was carried out as described previously (10). Briefly, 3,000 PFU of rHVT-CMV-*pmpD*, rHVT-EF-*pmpD*, and parental HVT were used to infect CEF cells seeded in T25 flasks. The cells were washed twice with PBS when the cytopathic effect occurred. Lysates of cells were subjected to 12% SDS-PAGE and electroblotted to polyvinylidene fluoride membranes, followed by 0.25% trypsin for 2 min. Membranes were incubated with the *C. psittaci* strain 6BC-specific polyclonal antibodies (diluted 1:100) previously prepared by our own lab, and then reacted with horseradish peroxidase-labeled goat-anti-chicken IgG (1:4,000) (Sigma-Aldrich, Shanghai, China). The PmpD-N glycoprotein bands were visualized using the enhanced DAB reagents (Tiangen, Beijing, China) according to the manufacturer’s instructions.

The expression and distribution of PmpD-N were determined in rHVT-CMV-*pmpD*-infected cells and rHVT-EF-*pmpD*-infected cells by immunofluorescence. First, CEF cells were infected with 3,000 PFU rHVT-CMV-*pmpD* and rHVT-EF-*pmpD*, respectively. The cells were probed with mouse anti-PmpD-N polyclonal serum of *C. psittaci* (previously prepared in our lab) and anti-HVT polyclonal serum (IVDC, Beijing, China) at a dilution of 1:100, and then overlaid with a mixture of goat anti-mouse IgG labeled with Alexa Fluor 488 (Invitrogen, Carlsbad, CA, USA) and goat anti-chicken IgY labeled with Alexa Fluor 568 (1:600 dilution) (Invitrogen, Carlsbad, CA, USA). Moreover, cell nuclei were stained with 1:10,000 dilution of 4,6-diamidino-2-phenylindole (DAPI) for 1 min or longer. Finally, the rinsed cover slips were mounted with Vectashield mounting medium (Vector Laboratories, Burlingame, CA, USA) and examined using a confocal microscope (Nikon, Tokyo, Japan).

### Measurement of Nitric Oxide (NO) and IL-6

HD11 cells were infected by 400 PFU rHVT-CMV-*pmpD* and rHVT-EF-*pmpD*, respectively. Cell culture supernatants were collected on day 1, 2, and 4 postinfection. OD values of supernatants were measured at 540 nm. NO production by activated HD11 cells was assessed as nitrite content in conditioned media using Griess reagent as illustrated previously (19). Sodium nitrite was used as the standard.

The infected cells treated above were also collected on day 1, 2, and 4 after inoculation. The cells were washed twice with PBS. Total RNA of cells on different day’s postinfection was extracted. The quantity of IL-6 was measured by relative quantitative real time RT-PCR, and the internal control gene was GAPDH as described previously (20).

### Statistical Analysis

Data were analyzed using the one-way ANOVA. A *P*-value of less than 0.05 was considered statistically significant.

## Results

### Generation of Recombinant HVT Expressing *pmpD*-N Gene Based on EF-1α Promoter

**Figure 1:**
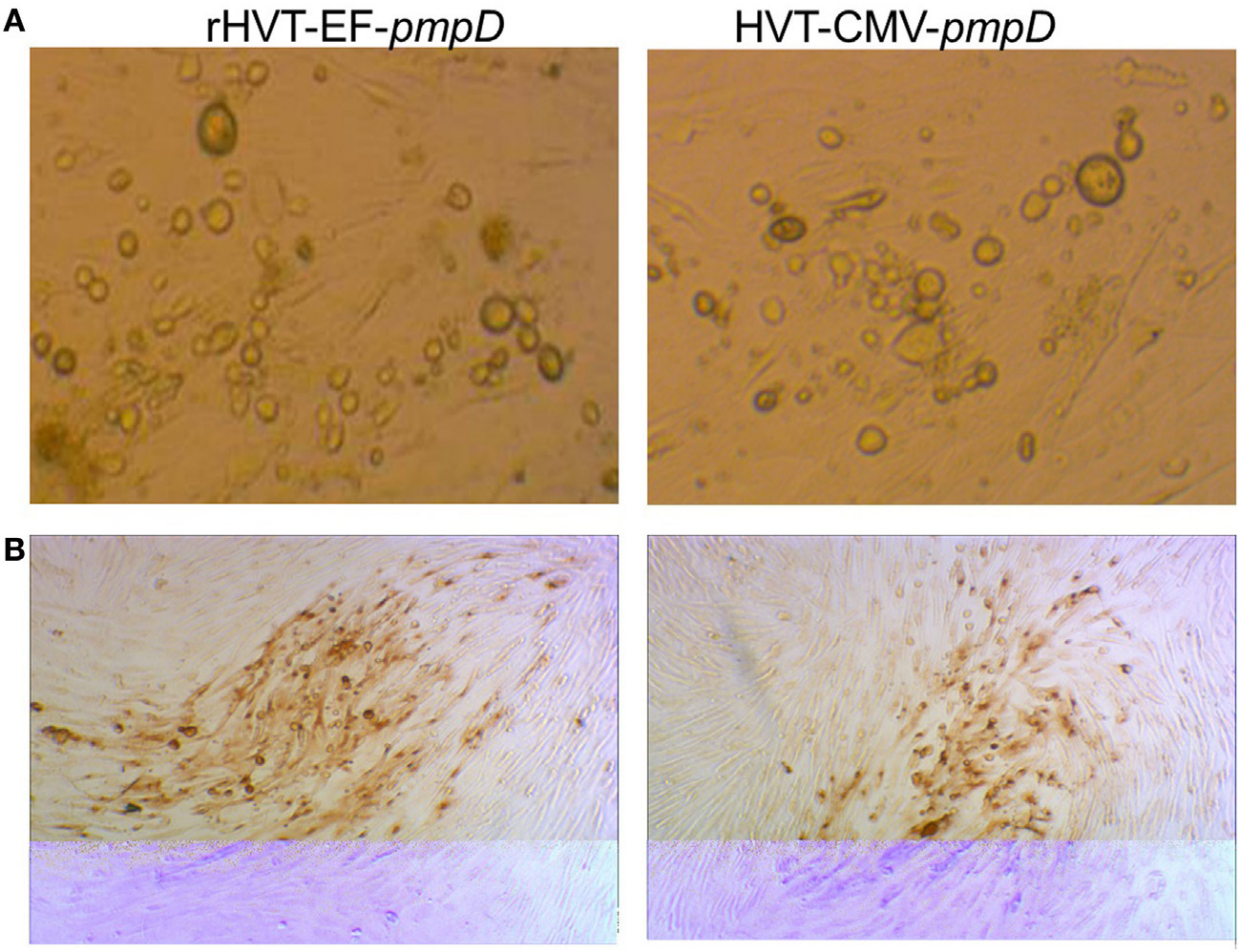
Cytopathic effect of rHVT-EF-*pmpD* and rHVT-CMV-*pmpD* on chicken embryo fibroblast (CEF) cells. **(A)** Morphology of the infected CEF cells induced by rHVT-EF-*pmpD* or rHVT-CMV-*pmpD* (magnifications 100×). **(B)** Immunohistochemical staining of CEF cells post inoculation with rHVT-EF-*pmpD* or rHVT-CMV-*pmpD* (magnifications 100×).

The *pmpD*-N gene with EF-1α promoter was successfully inserted into the HVT BAC region between UL45 and UL46. The recombinant HVT BAC DNA based on EF-1α promoter was transfected into CEF cells. Four days after transfection, typical plaques appeared in the CEF monolayers, these plaques being similar in size and morphology compared with those formed by rHVT-CMV-*pmpD* (Figure 1). The viral titration by staining plaques revealed no significant difference between rHVT-EF-*pmpD* and rHVT-CMV-*pmpD*.

### Expression of PmpD-N in rHVT-EF-*pmpD* Infected Cells

**Figure 2:**
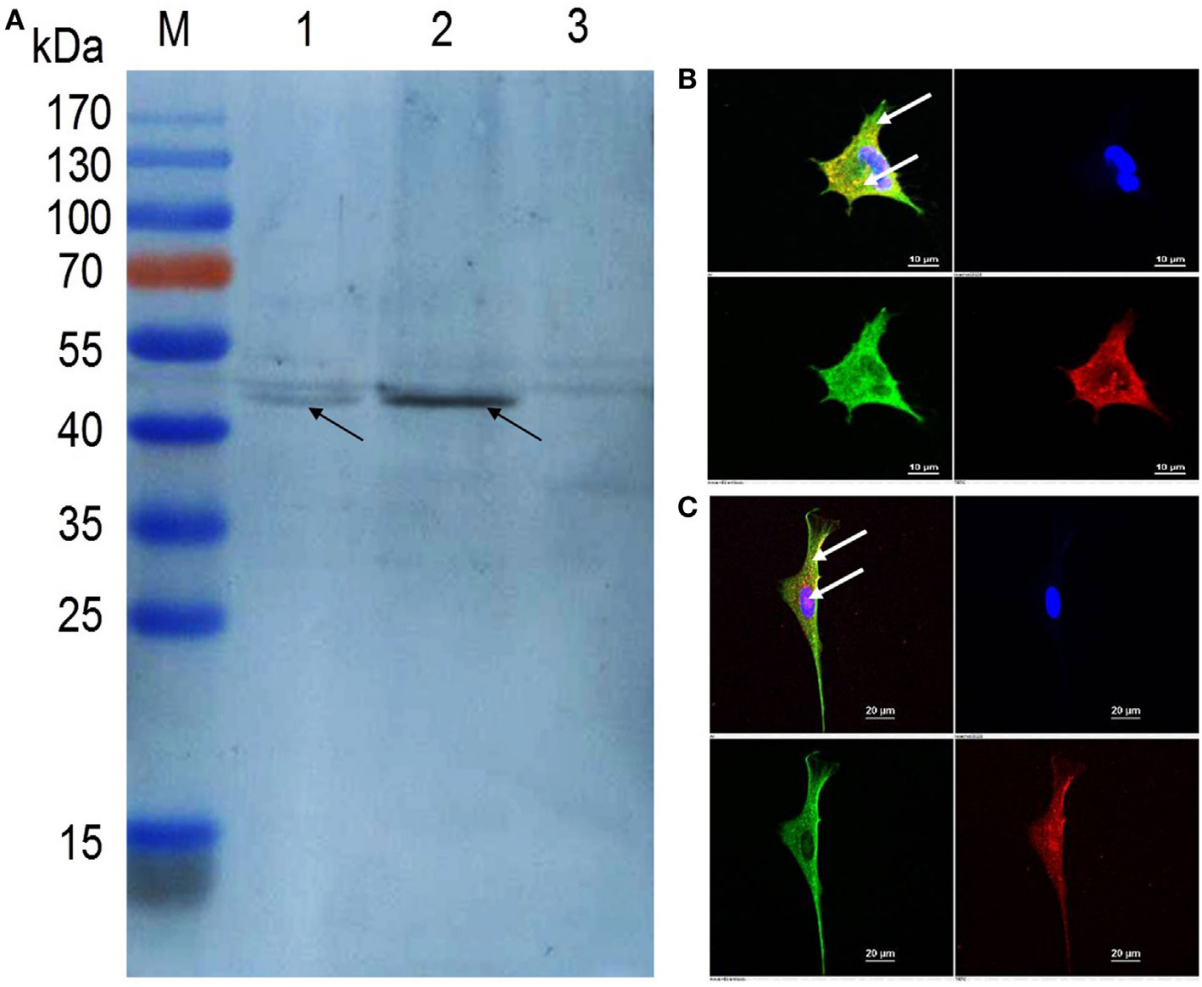
Confirmation of PmpD-N protein expression in herpesvirus of turkeys (HVT) vector by immunoblotting assay and indirect immunofluorescence. **(A)** The PmpD-N expression in rHVT-EF-*pmpD* was detected by immunoblot using *Chlamydia psittaci* strain 6BC-specific polyclonal antibodies. Lane M, pre-stained protein ladder; Lane 1, cell lysate post inoculation with rHVT-EF-*pmpD*; Lane 2, cell lysate post inoculation with rHVT-CMV-*pmpD*; Lane 3, cell lysate post inoculation with parental HVT. The black arrow indicates the approximate size of 43 kDa. **(B)** Indirect immunofluorescence analysis of PmpD-N expression in chicken embryo fibroblast (CEF) cells. CEF cells on glass coverslips were infected with rHVT-EF-*pmpD*, then incubated with mouse anti-PmpD-N polyclonal antibody of *C. psittaci* and chicken anti-HVT polyclonal serum, and then subsequently reacted with the goat anti-mouse IgG conjugated with Alexa Fluor 488 (green fluorescence, shown in the lower left panel) and goat anti-chicken IgY labeled with Alexa Fluor 568 (red fluorescence, shown in the lower right panel), respectively. Finally, cell nuclei were stained with diamidino-2-phenylindole (blue fluorescence, shown in the top right panel). The merged image is shown in the top left panel. The expression of the targeted protein is indicated by white arrows in the top left panel. **(C)** rHVT-CMV-*pmpD* control. CEF cells on glass coverslips were infected with rHVT-CMV-*pmpD*, and then the process of test and the panel meaning are the same as those shown in panel **(B)**.

To confirm the expression of the PmpD-N protein, CEF cells infected with rHVT-EF-*pmpD*, rHVT-CMV-*pmpD*, and parental HVT were analyzed by immunoblot and immunofluorescence. A 43-kDa band corresponding to the PmpD-N polypeptide was identified with polyclonal antibodies generated against *C. psittaci* 6BC strain by immunoblot analysis (Figure 2A). Under immunofluorescence microscopy, PmpD-N protein of rHVT-EF-*pmpD* and rHVT-CMV-*pmpD* was both expressed in the cytoplasm and on the cell surface (Figures 2B,C).

### One-Step Growth Kinetics

**Figure 3:**
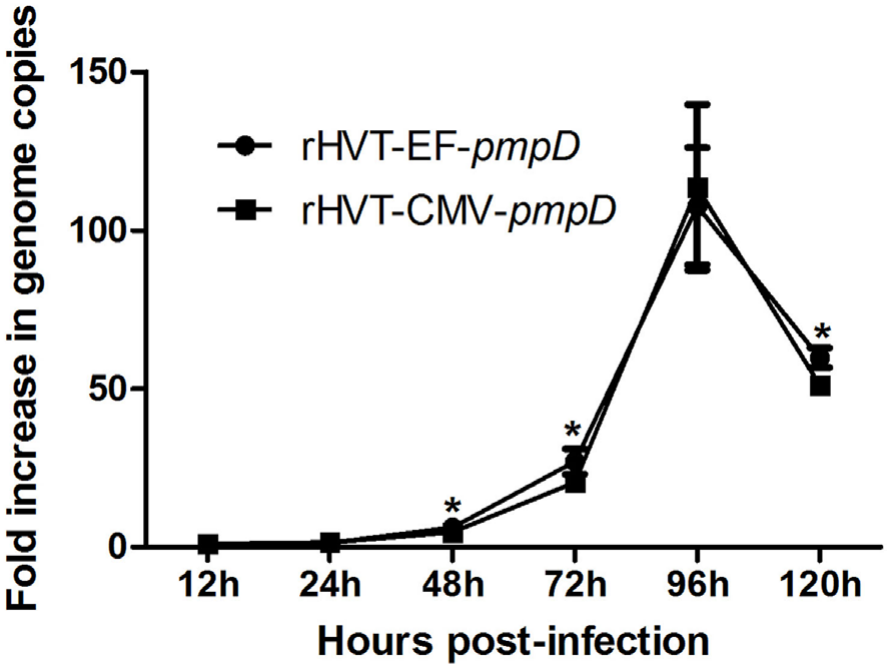
One-step growth kinetics of rHVT-EF-*pmpD* and rHVT-CMV-*pmpD in vitro*. The virus growth was calculated as the fold increase at different time points compared with the 12th hour postinfection. The asterisk indicates significant differences of the growth kinetics between rHVT-EF-*pmpD* and rHVT-CMV-*pmpD* (*P* < 0.05). The values were shown as means ± SD.

Growth trends of both recombinant viruses were similar as revealed by calculating the virus plaques at various times postinfection (Figure 3). Growth rates displayed a significant increase between 24 and 96 h, and a sharp decrease from 96 to 120 h. Except for 96 h postinfection, the growth rate of the rHVT-EF-*pmpD* was consistently higher than that of rHVT-CMV-*pmpD*, with a consequential difference appearing on 48, 72, and 120 h postinfection.

### Measurement of NO and IL-6

**Figure 4:**
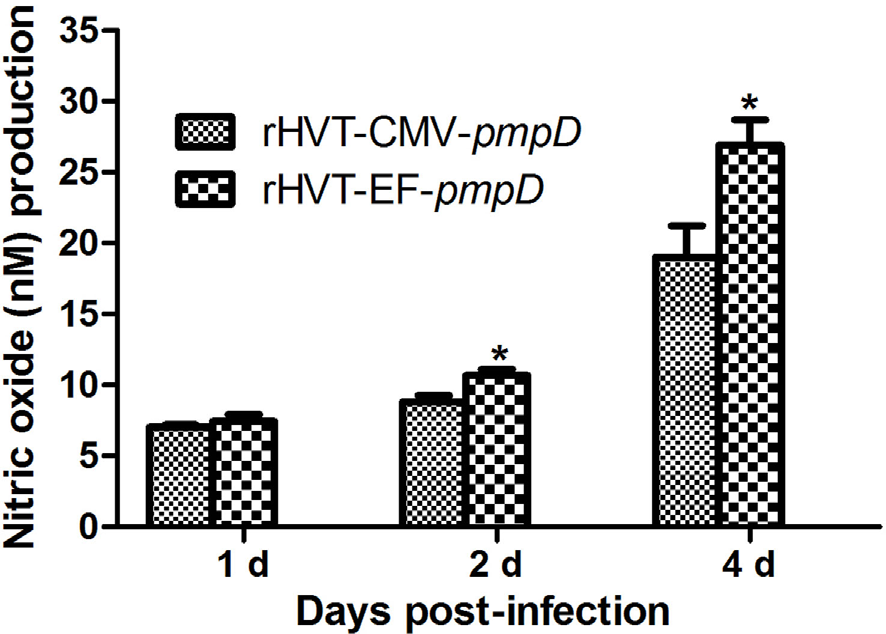
Nitric oxide (NO) responses of HD11 cells to rHVT-EF-*pmpD*. HD11 cells were subjected to 400 plaque-forming unit recombinant viruses for 1, 2, and 4 days. The supernatants were collected for NO analysis. Data were expressed as mean ± SE (*n* = 3) and were analyzed by Student’s *t*-test. Significance (*) was considered as *P* < 0.05 when compared with rHVT-CMV-*pmpD*.

**Figure 5:**
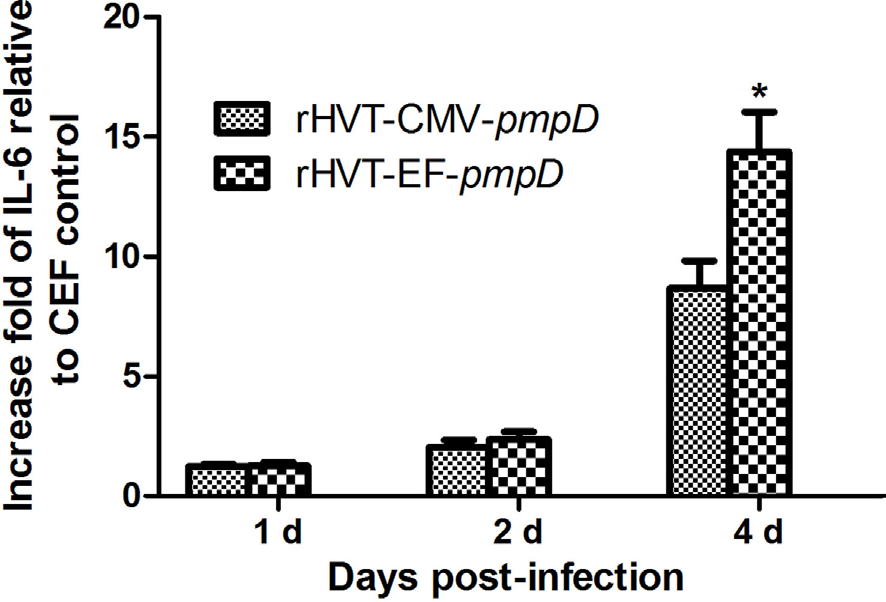
IL-6 responses of HD11 cells to rHVT-EF-*pmpD*. HD11 cells were subjected to 400 plaque-forming unit recombinant viruses for 1, 2, and 4 days. The cells were collected for IL-6 analysis using relative quantitative real time RT-PCR. The IL-6 increase fold of the recombinant viruses relative to chicken embryo fibroblast (CEF) cells was analyzed. Data were expressed as mean ± SE (*n* = 3) and were analyzed by Student’s *t*-test. Significance (*) was considered as *P* < 0.05 when compared with rHVT-CMV-*pmpD*.

The quantity of NO and IL-6 on HD11 cells infected by rHVT-CMV-*pmpD* and rHVT-EF-*pmpD* was assayed on days 1, 2, and 4. The NO production of rHVT-EF-*pmpD* was significantly higher than those of rHVT-CMV-*pmpD* on day 2 and day 4 (*P* < 0.05) (Figure 4). The IL-6 level of rHVT-EF-*pmpD* was apparently higher than that of rHVT-CMV-*pmpD* on day 4 (Figure 5).

## Discussion

*Chlamydia psittaci*, an obligate intracellular Gram-negative bacterium, often causes avian chlamydiosis and influenza-like symptoms in humans. At present, five serovars of *C. psittaci* have been found in chickens, which are genotypes B, C, D, F, and E/B (3, 21–23). The development of an efficacious vaccine against *C. psittaci* is needed as a high seropositivity rate was found in chickens (3–6). In this study, a recombinant HVT vaccine expressing PmpD-N based on EF-1α promoter was developed and was proved to be more efficient than that of CMV-based promoter.

To stimulate better immune protection, the constructed recombinant virus can deliver the target protein to the surface of the cell. Confocal analysis showed that the HVT vector could deliver PmpD-N protein to the surface of the cell in the rHVT-EF-*pmpD*, same as rHVT-CMV-*pmpD*. Plaque assay further showed that compared with rHVT-CMV-*pmpD*, the rHVT-EF-*pmpD* did not change the lesion status, plaque size and proliferation rate.

Macrophage, which belongs to phagocyte, participates in innate and cellular immunity in the body of animals. Macrophages display remarkable plasticity and can change their physiology in response to environmental cues. These changes can give rise to different populations of cells with distinct functions. Macrophages play an important role in monitoring, phagocytosis, uptake of antigens, secretion of various proinflammatory cytokines, and control and elimination of infections (24). In birds, macrophages play a central role in resistance to microbial infections and the pathogenesis of viruses, bacteria, and parasitic infections. Research shows that avian macrophages exposed to pathogens can be activated to produce proinflammatory cytokines, chemokines, reactive oxygen species (ROS), and NO, while ROS and NO are considered as antimicrobial arsenal that can control and remove pathogens. The HD11 cell line, which is obtained from avian macrophage like cell lines transformed by the avian encephalomyelitis virus, has the ability of phagocytosis and expressing Fc receptors and macrophage surface antigens (25). HD11 cells, as chicken macrophages, are widely used in the study of immune function *in vitro*. In this study, HD11 cells were used to analyze the macrophage that was impacted by the recombinant virus rHVT-EF-*pmpD* and rHVT-CMV-*pmpD*, respectively. Our results have shown that rHVT-EF-*pmpD* can significantly stimulate the macrophage to produce more proinflammatory cytokines IL-6 than rHVT-CMV-*pmpD* from day 2, and significantly stimulate the macrophage to produce more NO than rHVT-CMV-*pmpD* from day 4. Furthermore, the rHVT-EF-*pmpD* can stimulate the macrophage to kill more pathogens and promote more immune response than rHVT-CMV-*pmpD*.

In summary, the recombinant HVT expressing PmpD-N based on EF-1α promoter is more effective than our former constructed HVT based on CMV promoter *in vitro*. Further study is needed to analyze whether the rHVT-EF-*pmpD* could produce better protective immune response than rHVT-CMV-*pmpD in vivo*.

## Author Contributions

SL and CH designed the study. SL, WS, and XH performed experiments. SL, WZ, CJ, JL, and YS collected test data. SL and WS performed the date analysis and drafted the manuscript. CH and SE-A revised the manuscript. All the authors read and approved the final manuscript.

## Conflict of Interest Statement

The authors declare that the research was conducted in the absence of any commercial or financial relationships that could be construed as a potential conflict of interest.
